# “Gangrenous Finger” Proven to be Acute Gout

**Published:** 2015-06-08

**Authors:** Eric Gallagher, Todd Ruiter

**Affiliations:** ^1^Department of Orthopedic Surgery, Western Michigan University Homer Stryker MD School of Medicine; ^2^Borgess Medical Center, Kalamazoo, MI

**Keywords:** gout, uric acid, hyperuricemia, treatment, arthritis

**Figure:**
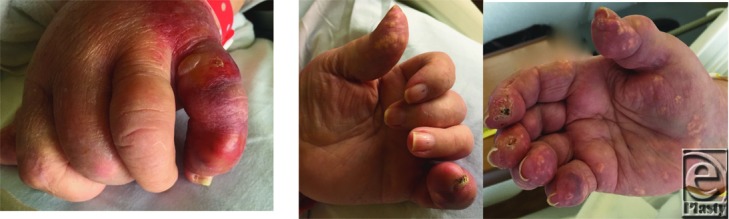


## DESCRIPTION

A 64-year-old woman was transferred from an outlying facility with a diagnosis of a “gangrenous finger.” She complained of 1 week of worsening left small finger pain, erythema, and swelling. Aspiration was performed upon transfer, and she received a diagnosis of acute gout. Oral colchicine and prednisone were begun with great improvement.

## QUESTIONS

1. **What is the pathophysiology of gout?**
2. **What are the clinical manifestations of gout?**
3. **What is the management for an acute gout flare?**
4. **What is the management for chronic gout?**

## DISCUSSION

Hyperuricemia is the most important risk factor for developing gout.^1^^,^^2^ There is an exponential increase in the frequency of gout with increasing serum uric acid levels over the threshold of solubility, which is approximately 6.8 mg/dL.^2^ Hyperuricemia is due to either overproduction or underexcretion of urate, with 85% to 90% due to underexcretion.^2^ Humans lack uricase, the enzyme that degrades urate to allantoin as the final step of purine nucleotide catabolism.^2^ We rely on renal excretion, mainly through URAT-1 transporter, to maintain homeostasis of urate. Hyperuricemia in combination with other risk factors leads to monosodium urate crystal deposition. Crystal deposition causes activation of neutrophils and an inflammatory reaction that results in an acute gout flare.

The clinical manifestations of gout are secondary to crystal-induced inflammation. Most often involving synovial structures and presenting as arthritis, tendonitis, and/or bursitis.^3^ Classically involving the first MTP, acute flares are more common in lower extremity than in upper extremity and typically involve only 1 joint.^2^^,^^3^ The hands are an uncommon presenting location but can be involved especially in patients with untreated chronic gout.^3^ Symptoms include a rapid onset of pain, swelling, and functional limitation of the involved structure.^2^^,^^3^ Erythema and local soft-tissue inflammation are often present and can be severe.^2^^,^^3^ Features of chronic gout include tophi, joint arthropathies, and joint deformity.^2^^,^^3^

The purpose of management of an acute gout flare is to decrease the length and intensity of symptoms. The mainstay of treatment is rest, ice, and nonsteroidal anti-inflammatory drugs (NSAIDs).^4^ There are no data to suggest that any specific NSAID or COX-2 inhibitor is more efficacious.^1^^,^^2^^,^^4^^,^^5^ NSAIDs are first-line therapy with rapid initiation, and high dosage being the most important factors.^2^^–^4 Colchicine, which works by reducing neutrophil migration, activation, and phagocytosis of monosodium urate crystals, is another option for acute flare.^4^^,^^5^ Low-dose colchicine is as effective as high dose and has lower frequency of adverse effects.^5^ In refractory cases or when NSAIDs and colchicine are contraindicated, oral, intramuscular, intra-alveolar, or intravenous corticosteroids can be used.^1^^,^^2^^,^^4^^,^^5^ Studies have shown corticosteroids to be as effective as NSAIDs.^4^ Overall, the data do not support that any of the treatment options are more efficacious; therefore, treatment should be based on individual cases.^5^

Management of chronic gout attempts to prevent acute flares and long-term sequelae.^1^ The recommendation is to maintain serum uric acid levels below 6 mg/dL.^1^^,^^2^^,^^5^ When initiating urate-lowering therapy, prophylaxis is essential because a high percentage of patients will experience a flare in the first 6 months of treatment without prophylaxis.^1^^,^^4^^,^^5^ The medications available for treatment are divided into uricostatic, uricosuric, and uricolytic agents. Uricostatic agents, allopurinol and febuxostat, decrease urate production through inhibition of xanthine oxidase.^1^^,^^2^^,^^4^ The mainstay of treatment is allopurinol; when contraindicated, febuxostat can be used.^1^^,^^2^^,^^4^ Uricosuric agents, benzbromarone, probenecid, and sulphinpyrazone, enhance renal clearance of urate through inhibition of URAT-1.^1^^,^^2^^,^^4^ These medications are used in a minority of patients when allopurinol is ineffective or contraindicated.^1^^,^^2^^,^^4^ Uricolytic drugs, rasburicase and polyethylene glycol–uricase, replicate the enzyme uricase. They are able to rapidly lower serum urate levels but have limited use due to potentially severe side effects and limited efficacy data.^1^^,^^2^^,^^4^ Overall, allopurinol is the first-line treatment, with other medications being used in patients who cannot tolerate allopurinol or have refractory cases.^5^

Gout can present a diagnostic dilemma due to the clinical similarities with septic arthritis and other inflammatory arthritides. Knowledge of the clinical presentation, pathophysiology, and management is essential to make an early diagnosis and initiate treatment. Our patient demonstrated the clinical features of both chronic gout and an acute gout flare. Initiation of colchicine and prednisone relieved her symptoms, but long-term therapy is critical to prevent recurrence.
